# Zygomycetes in Vesicular Basanites from Vesteris Seamount, Greenland Basin – A New Type of Cryptoendolithic Fungi

**DOI:** 10.1371/journal.pone.0133368

**Published:** 2015-07-16

**Authors:** Magnus Ivarsson, Jörn Peckmann, Anders Tehler, Curt Broman, Wolfgang Bach, Katharina Behrens, Joachim Reitner, Michael E. Böttcher, Lena Norbäck Ivarsson

**Affiliations:** 1 Department of Palaeobiology and the Center for Earth Evolution (NordCEE), Swedish Museum of Natural History, Stockholm, Sweden; 2 Department of Geodynamics and Sedimentology, Center for Earth Sciences, University of Vienna, Vienna, Austria; 3 Department of Botany, Swedish Museum of Natural History, Stockholm, Sweden; 4 Department of Geological Sciences, Stockholm University, Svante Arrheniusväg 8, Stockholm, Sweden; 5 MARUM–Center for Marine Environmental Sciences, University of Bremen, Bremen, Germany; 6 Geobiology Group, Geoscience Centre, Georg-August University, Göttingen, Germany; 7 Geochemistry & Isotope Biogeochemistry Group, Department for Marine Geology, Leibniz Institute for Baltic Sea Research (IOW), Warnemünde, Germany; 8 School of Natural Sciences, Technology and Environmental Studies, Södertörn University, Alfred Nobels Allé 7, Stockholm, Sweden; The University of Wisconsin—Madison, UNITED STATES

## Abstract

Fungi have been recognized as a frequent colonizer of subseafloor basalt but a substantial understanding of their abundance, diversity and ecological role in this environment is still lacking. Here we report fossilized cryptoendolithic fungal communities represented by mainly Zygomycetes and minor Ascomycetes in vesicles of dredged volcanic rocks (basanites) from the Vesteris Seamount in the Greenland Basin. Zygomycetes had not been reported from subseafloor basalt previously. Different stages in zygospore formation are documented in the studied samples, representing a reproduction cycle. Spore structures of both Zygomycetes and Ascomycetes are mineralized by romanechite-like Mn oxide phases, indicating an involvement in Mn(II) oxidation to form Mn(III,VI) oxides. Zygospores still exhibit a core of carbonaceous matter due to their resistance to degradation. The fungi are closely associated with fossiliferous marine sediments that have been introduced into the vesicles. At the contact to sediment infillings, fungi produced haustoria that penetrated and scavenged on the remains of fragmented marine organisms. It is most likely that such marine debris is the main carbon source for fungi in shallow volcanic rocks, which favored the establishment of vital colonies.

## Introduction

Subseafloor basaltic rocks have been recognized as an extensive microbial habitat harboring a substantial portion of microorganisms whose abundance, diversity and ecological role is far from understood [1,2]. Phylogenetic studies are rare due to sampling issues at great depths and conditions, but show a wide diversity among bacteria and archaea from both dredged samples [3–5], and deep drilled igneous rock [6–8]. As an alternative to phylogenetic methods investigation of fossilized material has proven useful. A diverse fossil record ranging from ichnofossils in volcanic glass [9] to fossilized endolithic communities in veins and vesicles of basalts [10–14] has been described. A majority of the recognized endoliths has been interpreted as fungal remains. Morphological characteristics such as repetitive septa, anastomosis, sporophores and central pores have been reported as well as chitin, a common constituent in fungal cell walls, and absent among prokaryotes [10,12,13].

Fungal colonization occurs relative early after the basalt has cooled, and prior to formation of secondary minerals like carbonates or zeolites [13,14]. Initially, the fungi cover the inner walls of the pore space with a biofilm from which hyphae and yeast structures grow and subsequently form mycelial-like networks. Associated with mycelia, fruiting bodies, spores and resting structures like sclerotia have been described, indicating that the fungi flourish in vital and sustainable colonies within the basalt [13,15]. The fungi have even been shown to exist in symbiotic-like relationships with prokaryotes, an absolute requirement for propagation in the basalts and for long term survival of heterotrophs in an igneous rock-hosted environment [14].

The subseafloor crust as a fungal habitat challenges previous conceptions of the deep biosphere being predominantly an ecological niche of chemoautotrophic prokaryotes. Still, we have only a fragmentary knowledge on the subseafloor fungal communities within igneous lithologies. A wide range of investigations including phylogeny, geochemistry and paleobiology, therefore, needs to be carried out to fully understand the role fungi play in these environments. Their ecological role needs to be established considering the geobiological impact fungi have in terrestrial environments, and their diversity needs to be investigated. Previously, Ivarsson et al. [12] interpreted subseafloor fungi as Ascomycetes or stem-group Dikarya. Based on morphological characteristics among zygospores, we here extend the fungal diversity to also include Zygomycetes, in dredged samples from the Vesteris Seamount in the Greenland Basin. We further show how fungi live on decomposing fragmented marine organisms from marine sediments introduced into the shallow vesicles, and also discuss Mn(II) oxidation and formation of Mn(III,IV) oxides in fungal spores.

## Samples and Methods

**Fig 1:**
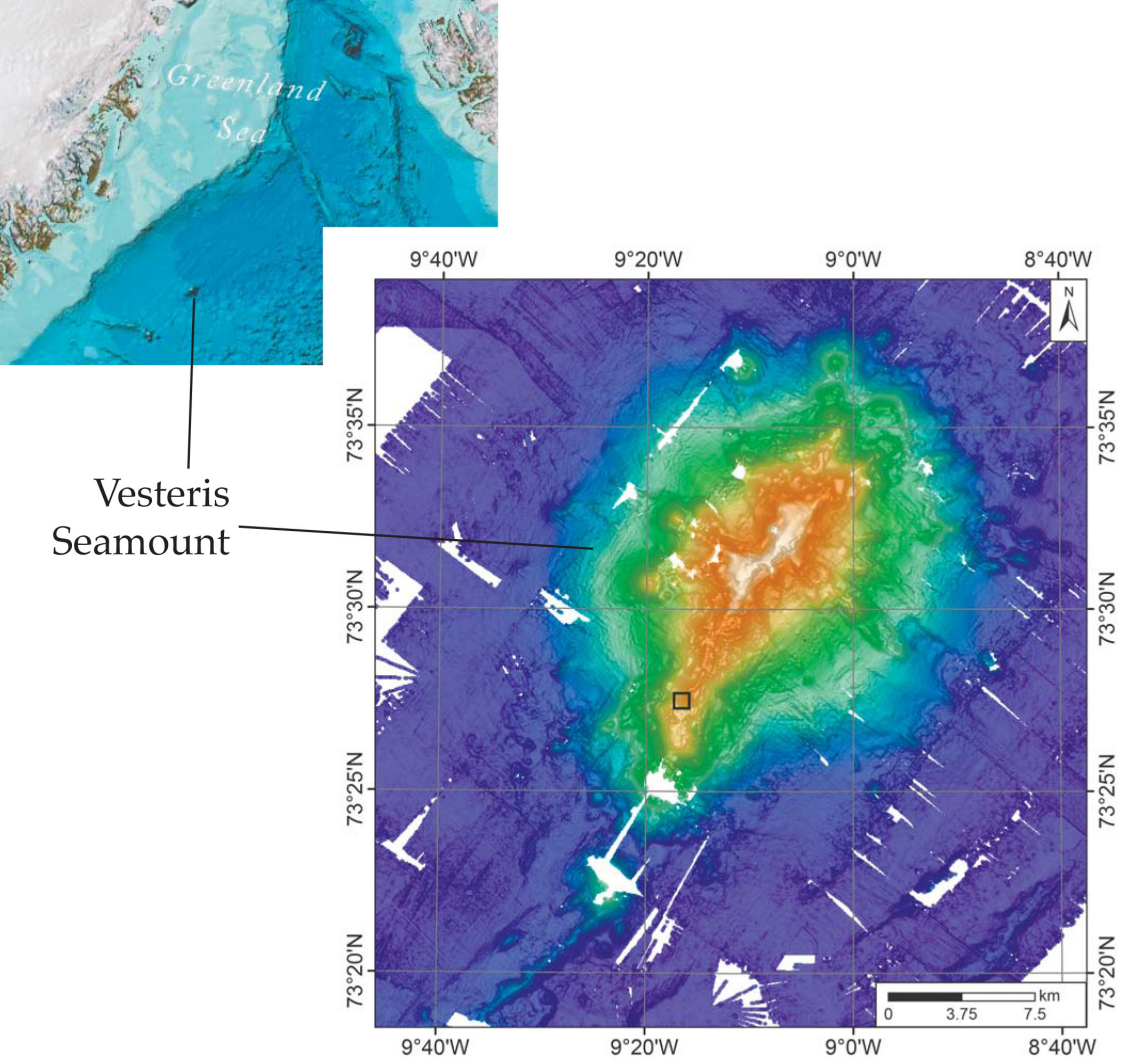
Map of Vesteris seamount. (A) Map of the northwestern part of the Atlantic showing the location of Vesteris Seamount in the Greenland Basin. Image reproduced from the GEBCO world map 2014, www.gebco.net. (B) Bathymetric map of Vesteris Seamount processed using combined multibeam data from RV Polarstern expeditions ARK II/4, ARK VII/1, ARKXVIII/1, and ARK XIX/4. The location from which the samples examined in this study were taken is marked by a black square.

The samples are from the Vesteris Seamount, approximately 300 km north of the island of Jan Mayen (Fig 1) and were retrieved by TV-guided grab during expedition ARKVII/1 by RV Polarstern in June/July of 1990 [16]. The sample was not collected in a protected area and specific permissions were not required for sampling. The field studies did not involve endangered or protected species. The investigated material is deposited in the collections of the Geoscience Museum of the University of Göttingen, Germany (GZG-PB.4041–4045), and accessible by the general public.

Vesteris Seamount is a young singular volcanic edifice resting upon 44 Ma old basement in the >3000-m deep central Greenland Basin. Vesteris Seamount is composed of highly alkaline lavas of alkalibasaltic to basanitic/tephritic compositions, consistent with small extents of mantle melting due to great lithospheric thickness [17]. Vesteris Seamount is volcanically active and the age of the tephritic rocks, in which the fossils are found, is 0.5 to 0.65 Ma, as determined by Ar-Ar dating of kaersutite phenocrysts [18]. Video sled seafloor mapping has indicated very little sediment accumulation in the summit area, which is densely populated by sponges and shows evidence for spotty low-temperature hydrothermal activity [19].

The samples were collected at 73°27.43’N, 09°16.34’W and 727 m water depth [19] from the southern part of a 15 km long, crescent-shape, NE-SW trending crest developed in the summit area of the volcano. The sample location is near the top of a steep south-facing slope leading to a lower-tiered section of the southernmost crest (Fig 1). The seafloor there is composed of scoracious lava decorated with small sponge-crinoid mounds and featuring minor accumulations of sandy mud in small depressions [19]. The samples studied here are identical to samples of pillow basalt studied by Reitner et al. [20] that had been erroneously related to another sampling site of expedition ARKVII/1 on the Kolbeinsey ridge.

The samples were prepared as 11 large (10x15 cm), 8 medium large (7.5x10 cm), and 12 small (4.8x2.8 cm) thin sections. Mineralogy and fossils were studied by light microscopy, electron micro-probe analyzer, and Raman spectroscopy. Additionally, oxidized Fe and Mn contents as well as the geochemical major, minor and trace element composition were analyzed.

### Bulk rock analyses

The rim and core parts of one specimen were separated and the sieved grain size fraction smaller than ~63 μm was further used. Bulk elemental analysis was carried out via X-ray fluorescence spectroscopy at ICBM, University of Oldenburg (Philips PW 2400 spectrometer; [21]). Reducible Fe(III) and Mn(IV) were extracted via buffered sodium-citrate-acetic-dithionite solution [22]. Extracted iron contents were analyzed by spectrophotometry and manganese with atomic absorption spectroscopy (Perkin Elmer spectrometer) after appropriate dilution. Total carbon contents (TC), total nitrogen (TN) and sulfur (TS) was determined via elemental analysis (Fisons EA), and total inorganic carbon (TIC) by coulometry. The total organic carbon content was calculated as the difference between the TC and TIC contents. Total reducible reduced inorganic sulfur (TRIS) was extracted by means of and selective acid extraction with hot acidic Cr(II) chloride solution, the evolved hydrogen sulfide trapped in zinc acetate solution [23] and measured spectrophotometrically according to Cline [24]. Mercury contents were analyzed with atomic absorption spectroscopy [25]. Water-extractable contents of chloride were analyzed via ion chromatography (Waters ion chromatography). Figured samples are deposited in the specimen collection of the Geoscience Museum of the University of Göttingen, Germany.

### Electron Micro-probe Analyzer

Electron microprobe analyses were carried out at the University of Kiel using a Jeol JXA 8900R instrument. Analytical conditions were 15 kV accelerating potential, 20 nA probe current, and 1 μm beam diameter. Natural minerals or synthetic materials were used for calibration, and data were corrected for matrix effects by the phi-rho-z method. Back-scatter electron (BSE) images were used to select different phases for quantitative analysis.

### Raman spectroscopy

The analyses were performed at the Department of Geological Sciences, Stockholm University, with a confocal laser Raman spectrometer (Horiba instrument LabRAM HR 800), equipped with a multichannel air-cooled (-70°C) 1024 x 256 pixel CCD (charge-coupled device) array detector. Acquisitions were obtained with an 1800 lines/mm grating. Excitation was provided by an Ar-ion laser (= 514 nm) source. A low laser power 0.05 mW at the sample surface was used to avoid laser induced degradation of the sample. A confocal Olympus BX41 microscope was coupled to the instrument. The laser beam was focused through a 100x objective to obtain a spot size of about 1 μm. The spectral resolution was ~0.3 cm^-1^/pixel. The accuracy of the instrument was controlled by repeated use of a silicon wafer calibration standard with a characteristic Raman line at 520.7 cm^-1^. The Raman spectra were achieved with LabSpec 5 software. The samples are poorly crystalline and to avoid sample distortion Raman spectra were recorded using a low excitation power. Even though the obtained bands therefore appear with a weak intensity, characteristic peaks can be distinguished for the identification of phases.

## Results

**Fig 2:**
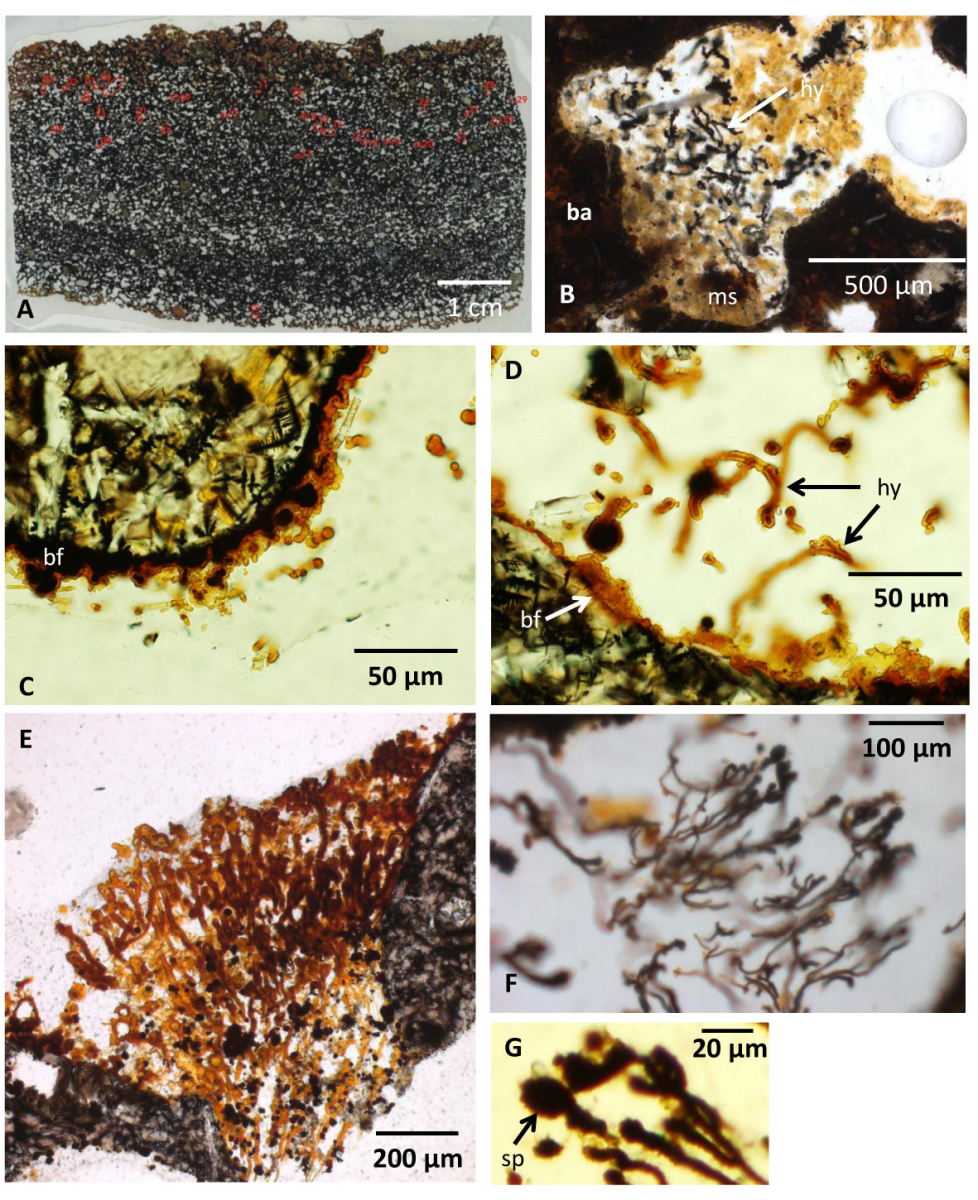
Microphotographs of the vesicular basanite and fungal communities within. (A) Thin section showing the vesicularity of the pillow lava. (B) A vesicle partly filled with marine sediments. Fungal hyphae occur in association with the sediment. (C) Fossilized biofilm lining the vesicle walls with spherical structures and a few protruding filaments. (D) Fossilized biofilm on the vesicle wall from which hyphae protrude. (E) Hyphae of the first bush-like type with a directed growth occupying the void in between two vesicles. (F) Hyphae of the first bush-like type with a directed growth and oval terminal swellings. (G) Close-up of oval terminal swellings. Legend for all figures: ba, basanite; ms, marine sediments; hy, hyphae; bf, biofilm (fossilized); sp, sporophore/spore; an, anastomose; cs, central strand; zph, zygophore; pr, progametangia; ga, gametangia; mzy, maturing zygote; zy, zygote; di, diatome, ra, raphe; ar, areolaes; fmo, fragmented marine organism; ha, haustorium.

The samples consist of highly vesicular basanite with vesicles ranging up to several millimeters in diameter (Fig 2A). The rock is composed of clinopyroxene and sparse olivine microphenocrysts as well as plagioclase phenocrysts in a glassy mesostasis. The glass has basanitic composition, with silica contents ranging between 44.4 and 46.1 wt.%, and Na_2_O+K_2_O between 5.9 and 6.8 wt.%. The analyses give 10–12 wt.% normative olivine, indicating that compositions are almost tephritic (i.e., <10% normative olivine [26]). The bulk rock compositions (Table 1) are clearly basanitic and similar to fresh volcanic rock samples from the same TV-grab station reported by Haase and Devey [17]. A geochemical comparison of crustal and core parts of one specimen show that the crustal part is relatively enriched in extractable Fe(III), Mn(IV), and chloride. The crustal part also contains more TOC, As, Pb and Zn. The contents of Al, Si, Ti, Ca and Mg show minor differences as well, indicating a slightly in-homogeneous main mineral distribution. When compared to the fresh rock composition (Table 1), the altered samples reveal slight losses of Mg and Si, accompanied by gains in Al and Fe. These trends are typical for low-temperature alteration of basic volcanic glass.

**Table 1:** Bulk rock compositions of the outer margin and the core of a basanite flow in comparison with fresh material from the same grab (21891–4).

	Margin	Core	Fresh*
SiO_2_ (wt.%)	35.5	39.2	42.5
TiO_2_ (wt.%)	3.6	2.7	2.6
Al_2_O_3_ (wt.%)	13.7	13.5	12.9
Fe_2_O_3_ (wt.%)	14.2	11.6	12.1
MnO (wt.%)	0.35	0.16	0.18
MgO (wt.%)	11.3	11.9	14.2
CaO (wt.%)	12.6	12.2	12.2
Na_2_O (wt.%)	1.61	2.16	2.09
K_2_O (wt.%)	0.96	1.21	0.95
P_2_O_5_ (wt.%)	0.52	0.40	0.39
Total (wt.%)	94.3	95.0	100.1
TOC (wt.%)	0.20	0.09	n.d.
TIC (wt.%)	0.13	0.09	n.d.
TN (wt.%)	0.06	0.03	n.d.
S-T (wt.%)	0.33	0.26	n.d.
S-TRIS (wt.%)	0.01	0.02	n.d.
Cl* (wt.%)	0.29	0.15	n.d.
Fe-T (wt.%)	9.9	8.1	n.d.
Fe* (wt.%)	2.57	0.77	n.d.
Mn-T (wt.%)	0.27	0.16	n.d.
Mn* (wt.%)	0.13	0.03	n.d.
As (mg/kg)	57	14	n.d.
Ba(mg/kg)	295	370	353
Co (mg/kg)	72	50	58
Cr (mg/kg)	624	843	747
Cu (mg/kg)	236	240	81
Hg (μg/kg)	8	6	n.d.
Ni (mg/kg)	207	219	285
Pb (mg/kg)	7	2	2
Rb (mg/kg)	14	21	22
Sr (mg/kg)	465	512	507
V (mg/kg)	459	368	n.d.
Y (mg/kg)	41	27	23
Zn (mg/kg)	110	86	n.d.
Zr (mg/kg)	239	187	180

*Data from [17]

n.d. = not determined.

The vesicles are empty or partly to completely filled with sediments (Fig 2B). The vesicle walls are lined with a crust that varies from a few μms to ~20 μm in thickness (Fig 2C and 2D). The crust has an irregular morphology being partly smooth and flat, partly botryoidal, and partly intermixed with spherical structures ranging from ~10 to ~50 μm in diameter. Yellowish filaments with diameters ranging from ~10 to ~20 μm and lengths of several hundred μms protrude from the crusts (Fig 2C and 2D). The filaments either occur as single filaments or exist in complex networks that may fill large portions of the vesicles (Fig 2E and 2F). Most filaments have been embedded in epoxy from the thin section preparation, but filaments also occur in the marine sediments (Fig 2B).

The filaments show a high variation in morphology, but two types can be discriminated. The first type is 10–20 μm in diameter and hundreds of μm in length, branches frequently, and has a bush-like appearance with a mutual growth direction (Fig 2E and 2F). Branching usually results in filaments of similar diameter as the original filament. These filaments have terminal swellings, often oval in shape, with a dark anisotropic appearance compared to the yellowish isotropic filaments. The club-shaped terminal swellings are ~20 μm in diameter (Fig 2G).

**Fig 3:**
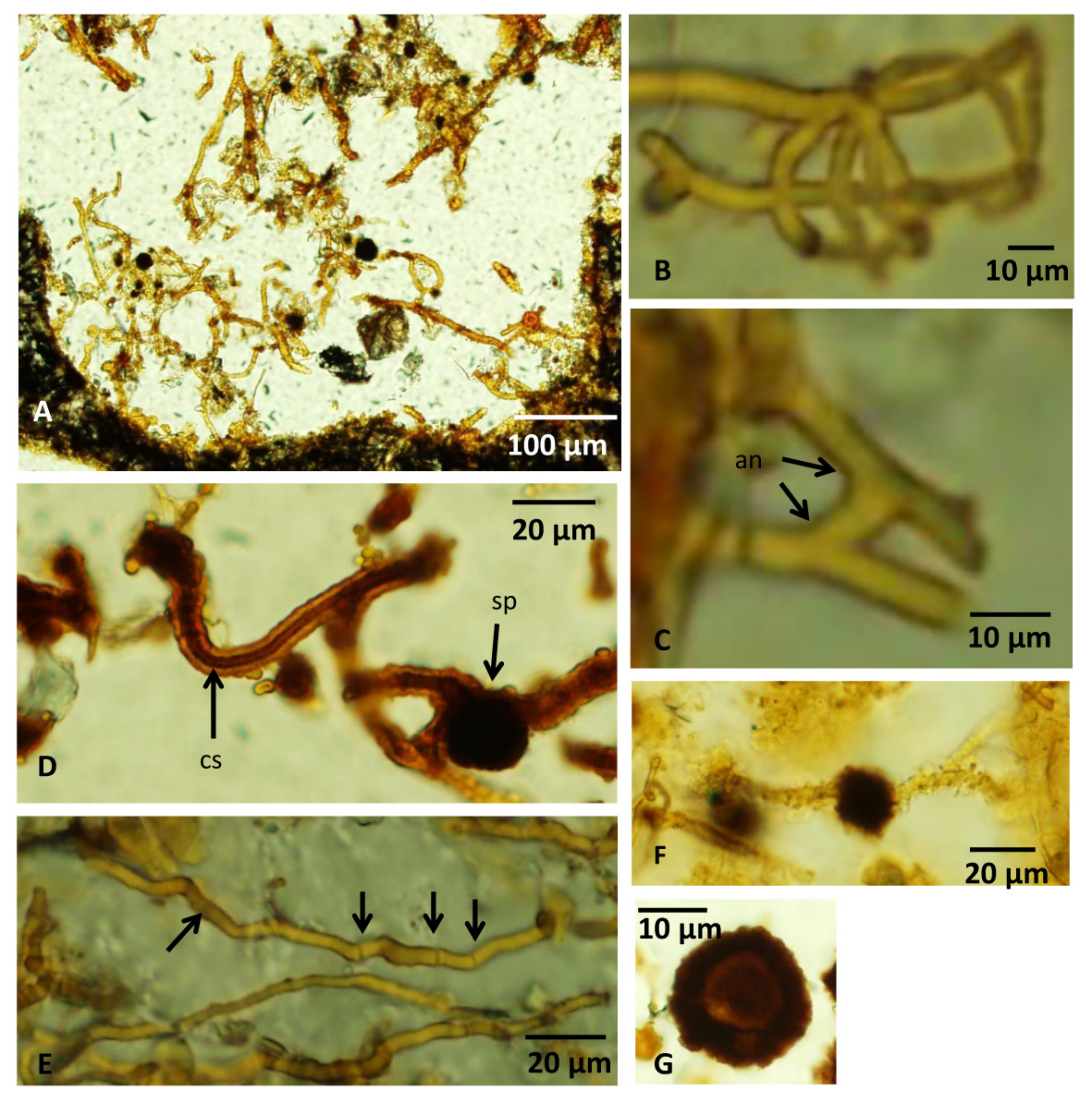
Microphotographs of fungal hyphae and spores. (A) Overview of a vesicle filled with hyphae of the second type without a mutual directed growth. (B) Branched hyphae. (C) Hyphae with an anastomose. (D) A hyphae with a central strand. A spherical spore structure is seen on another hypha. (E) Irregularly spaced septa along a hyphae marked with arrows. (F) A dark, swollen, anisotropic spore structure along a hyphae. (G) A spherical spore with a distinct core with carbonaceous matter according to Raman analyses. For legends see caption of Fig 2.

**Fig 4:**
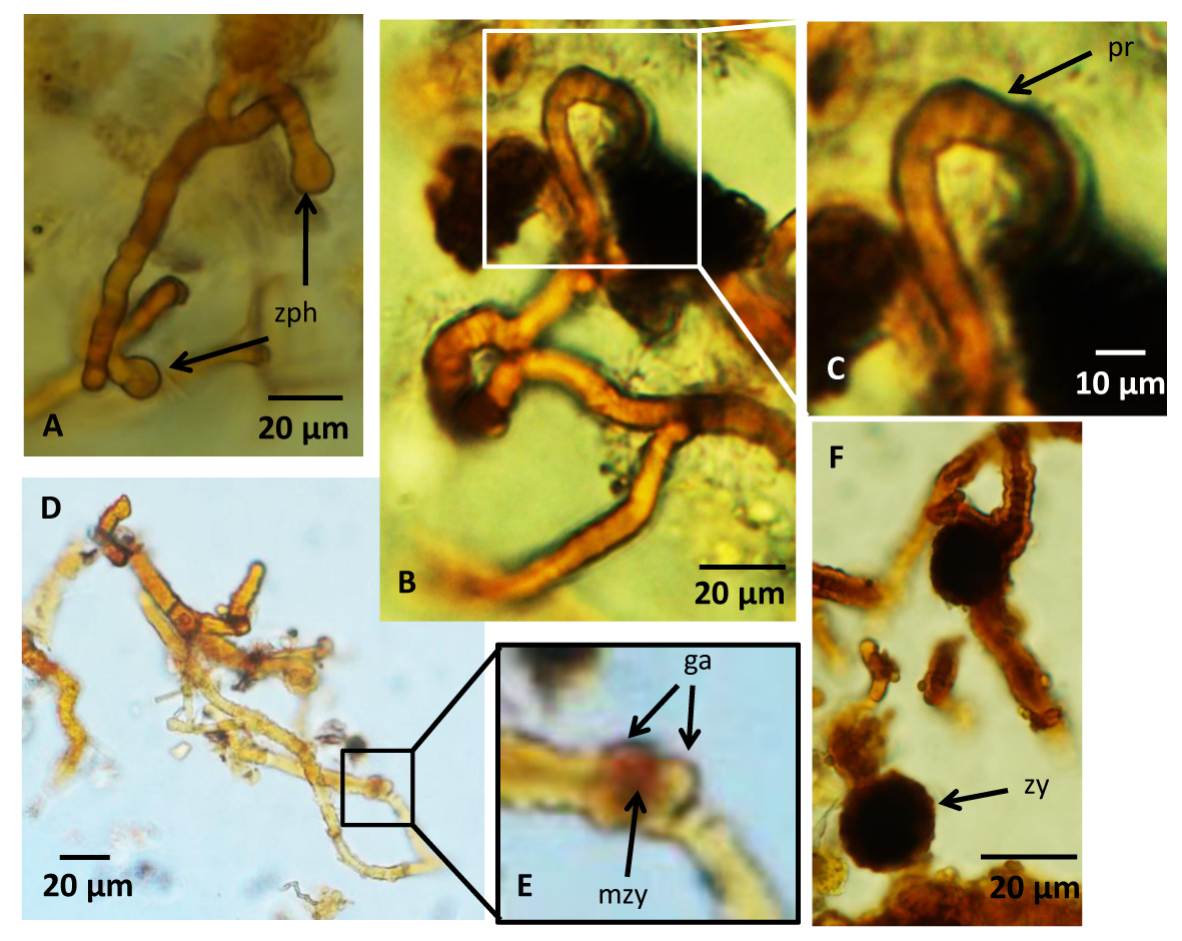
Microphotographs of zygospores and a Zygomycetes reproduction cycle. (A) Two zygophores approaching each other. (B,C) Fusion of two zygophores to form progametangia. Note how the zygophores are tightly appressed parallel to each other while fusing to form the progametangia. (D,E) Gametangia are separated by septa and a structure develops in between them that could represent early stage of zygote formation with fusion wall beginning to degrade. (F) Image showing a swollen, dark structure with a warty appearance that represents the last stages of the zygospore formation, the zygote. For legends see caption of Fig 2.

The second type has no common direction of growth, but exists in complex networks (Fig 3A). Their diameter ranges from ~5 to ~20 μm and their length can be several hundred μms. They branch frequently and anastomoses between branches occur (Fig 3B and 3C). These filaments either lack septa and have a distinct central strand, approximately half of the outer diameter of the filament (Fig 3D), or have irregularly spaced septation and lack a distinct central strand (Fig 3E). This second type is characterized by dark, anisotropic spherical swellings with an ornamented exterior and diameters of ~20 μm (Fig 3D, 3F and 3G). These swellings differ from the swellings of the first filament type in that they are not terminal swellings but occur along filaments. Some contain a core that is distinct from the margins (Fig 3G). In filaments relative close to each other swollen tips occur (Fig 4A), and when these are in direct contact with each other, a thick septum-like structure is developed (Fig 4B and 4C). In filaments where this process seems to have proceeded further, tips with a distinct mirrored morphology of swollen outgrowths occur (Fig 4D and 4E). Similar morphologies are also seen closely associated with filament loops that either are formed from two filaments located almost parallel to each other or represent a loop on the same filament (Fig 4D and 4E).

**Fig 5:**
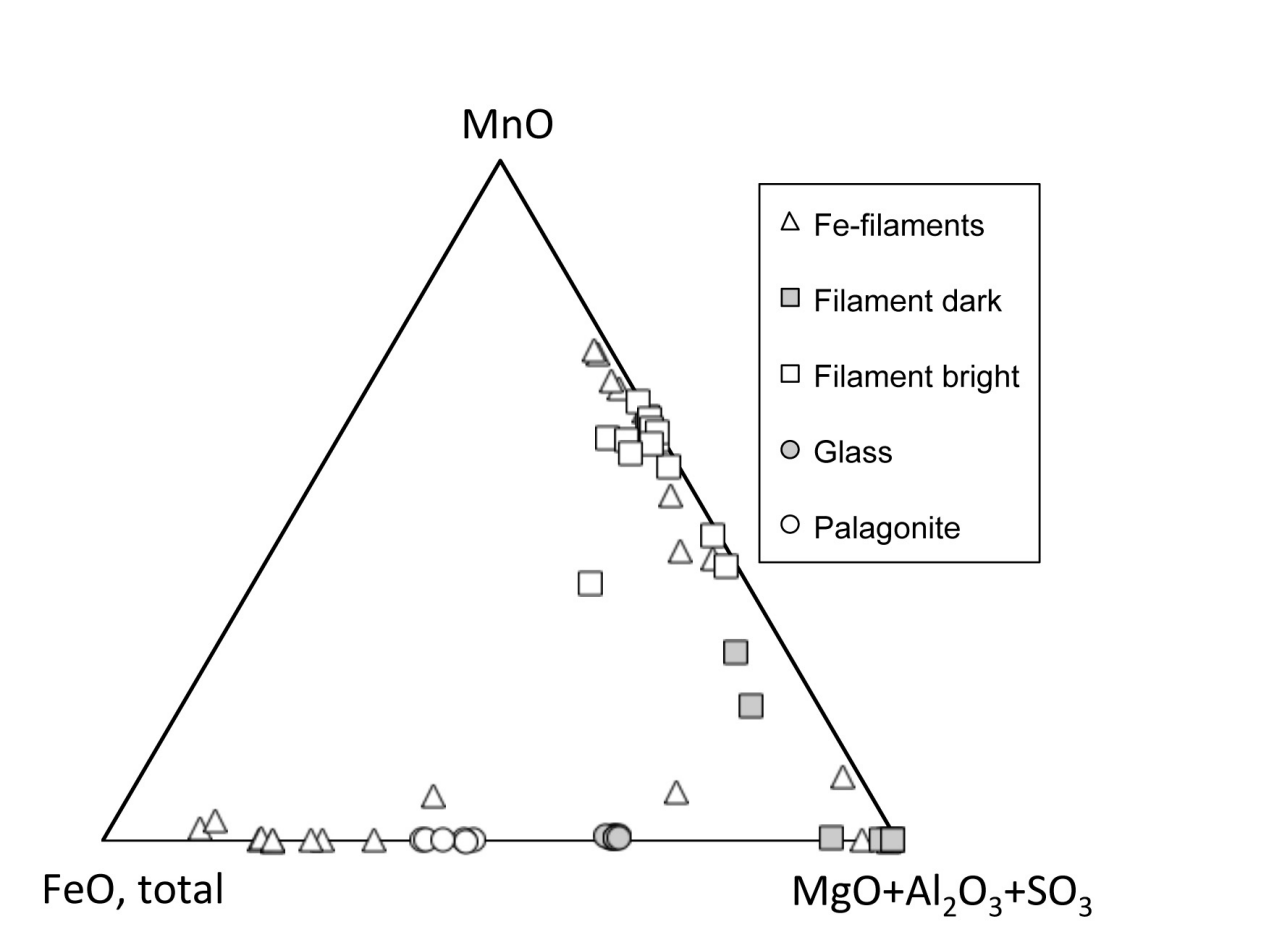
Electron micro-probe data. Overview of compositional differences between the different secondary mineral formations in comparison with that of fresh glass. The squares represent analyses of the simple filament type with BSE-bright Mn-rich interior strands. The triangles represent the filaments with more complex zoning, including Fe-rich outer parts.

Electron micro-probe analyses indicate that the yellowish filaments are compositionally zoned (Fig 5). They either have BSE-bright cores that are composed primarily of Mn oxide, with approximately 10 wt% of each MgO and Al_2_O_3_ present and elevated contents of Cu (around 2 wt.%). These cores are surrounded by BSE-dark zones that have subequal amount of MgO and Al_2_O_3_ (20–25 wt.%), 5–9 wt.% SO_3_, variable MnO, and invariably low FeO contents. The composition of these zones resembles that of motukoreaite, a hydrotalcite-group mineral known to occur in oxidative alteration of seafloor basalt [27]. Another type of zoning is more complex and shows iron-rich outer zones around central parts that often have finely intergrown or rhythmically layered domains with highly variable BSE-brightness and chemical composition. These interior parts appear to contain phases resembling the ones from the simply zoned filaments. The compositions of all filaments are distinct from that of palagonite, the product of abiotic alteration of the basanitic glass (Fig 5).

**Fig 6:**
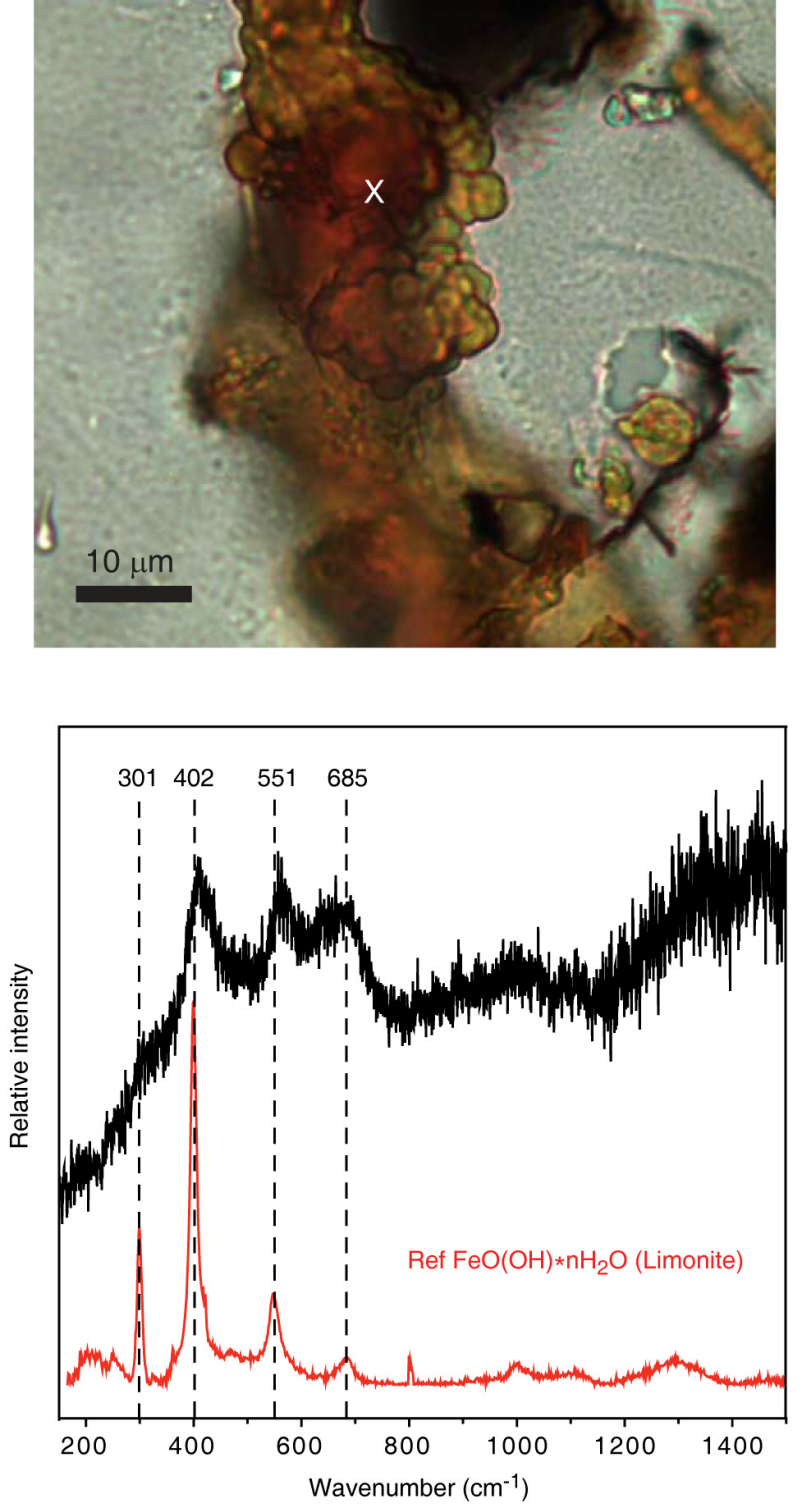
Raman spectrum of a filament. The obtained spectrum is identified as FeO(OH) * nH_2_O (limonite) after comparison with RRUFF reference spectra from Downs (2006) with typical bands at 301, 403, 551 and 685 cm^-1^. The position were the spectrum was taken is indicated by a ‘x’ in the photomicrograph.

**Fig 7:**
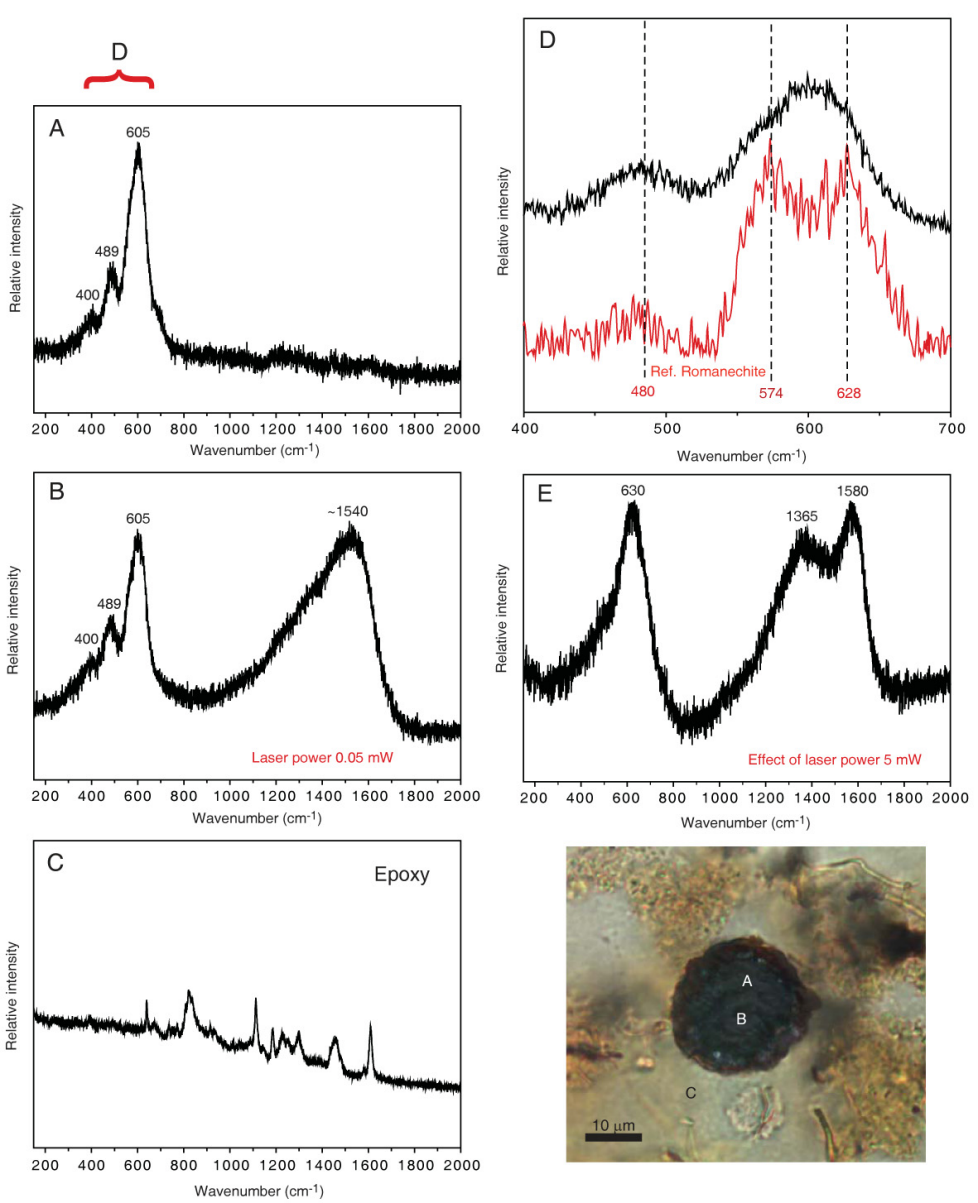
Raman spectra of the dark spherical structures. The obtained spectra are identified after comparison with RRUFF reference spectra from Downs (2006). The analyses were made using a low laser power (0.05 mW) at the positions in the sample that is indicated by the letters A to C in the photo. The corresponding spectra measured in the range 150 to 2000 cm^-1^ are given in A-C. The spectra in A and B display bands at 400, 489 and 605 cm^-1^ that suggest a Mn oxide phase and in spectrum B an additional band around 1540 cm^-1^ can be recognized which indicates carbonaceous material. (C) Representative spectrum of the epoxy used to make the thin sections to support the assertion that the Raman spectra of the spherical structures are from the rock sample. (D) The 605 cm^-1^ band consists of overlapping bands and spectra best match a romanechite-like manganese dioxide phase characterized by broad bands at 480, 574 and 628 cm^-1^; scale of the x-axis has been adjusted to the range between 400 and 700 cm^-1^. (E) Influence of a higher excitation power (5 mW) with the resultant transition of the romanechite-like phase to a todorokite-like manganese dioxide phase (the band at 630 cm^-1^) and the carbonaceous phase to slightly more ordered material (with bands centered at about 1365 and 1580 cm^-1^).

Raman analyses, after comparison with RRUFF reference spectra from Downs [28], indicate that the Fe-oxide phase in the filament is limonite (FeO(OH) * nH_2_O) with typical bands at 301, 403, 551 and 685 cm^-1^ (Fig 6). The black swellings in both types of filaments have a chemical composition corresponding to Mn-oxides but with a significant Mg content, and, thus, are distinctly different from the yellowish filaments they are associated with (Fig 5). Raman spectra of the dark Mn-oxide swellings best match a romanechite-like manganese dioxide phase characterized by broad bands at 480, 574 and 628 cm^-1^, according to RRUFF reference spectra from Downs [28] (Fig 7). In swellings of the second type of filaments a high content of carbonaceous matter (CM) is indicated by a band around 1540 cm^-1^ in the Raman spectra. The CM is located in specific areas in the core of the swellings, but is also detected at the margins of the swellings. Spectra for the surrounding glue were obtained to exclude contamination. The chemical composition of the crust is similar to the composition of the filaments.

**Fig 8:**
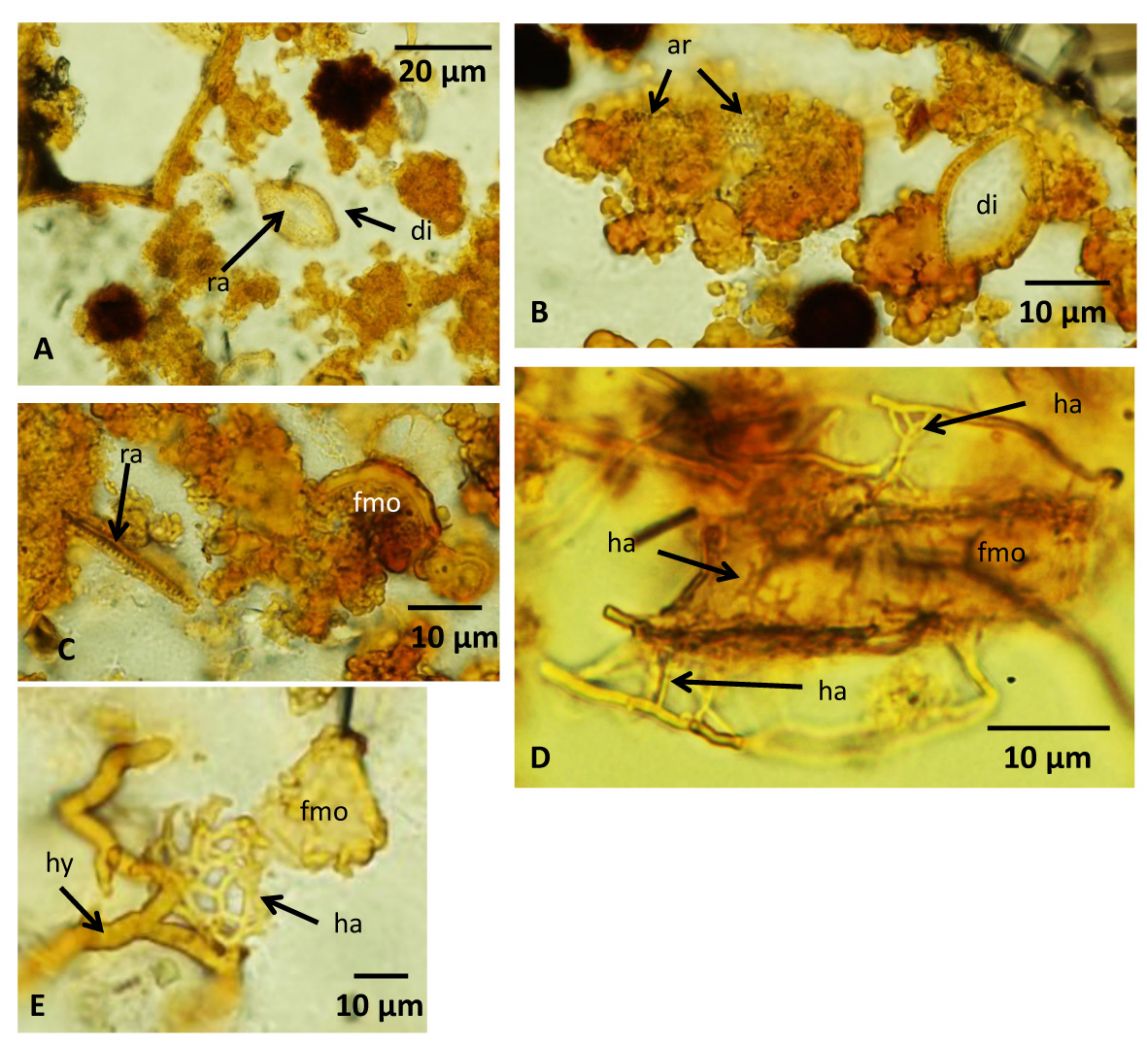
Microphotographs of fragmented marine organisms and hyphae forming haustoria. (A) Marine sediments with remains of a likely diatom with a visible raphe across the center. (B) Remains of diatoms in marine sediments. The left diatom is overgrown by fungi and irregular organic matter, but structures resembling areolaes are visible through the organic cover. (C) Remains of a raphe and unidentified fragmented marine organism in marine sediments. (D) An unidentified fragmented marine organism surrounded by hyphae that form protruding haustorium penetrating the fragment. Haustorium is seen with the fragment as well. (E) A hyphae that forms a mycelial haustorium in association with the remains of a fragmented marine organism. For legends see caption of Fig 2.

The marine sediments enclosed in much of the vesicles contain many fragmented marine microorganisms of various morphologies, sizes and degree of decay. Most of these fragments cannot be identified, but likely remnants of diatoms with areolae, striae and a raphe were recognized (Fig 8A–8C). When the filaments of the second type occur in close contact with or within the marine sediments, they attach to and enclose the fragments of marine organisms (Fig 8B and 8D). At contact with the fragmented organisms the filaments divide in several smaller filaments with diameters of ~1 μm and less (Fig 8D and 8E) that penetrate the fragments. These small filaments branch frequently and anastomoses between branches occur, forming an intricate small-scaled network. Fragments with abundant filaments are highly degraded and an irregular mass associated with the filaments surrounds them.

## Discussion

### Biogenicity and fungal interpretation

We argue for a biogenic origin of the filaments based on four criteria. 1) *Geological context*. Subseafloor basalts have been recognized as a considerable microbial habitat, perhaps Earth´s largest [1]. Fossilized endolithic communities in volcanic glass and in veins and vesicles in the basalt shows that life is widely distributed spatially and with depth [9,11,29,30]. A majority of the fossilized endoliths represent fungi, thus, the subseafloor oceanic crust represent an environment inhabited by both prokaryotes and eukaryotes [10,12–14].

2) *Syngenicity and indigenousness*. The filaments originate from the crust of secondary, newly formed minerals that lines the inner vesicle walls. There are smooth and undisturbed transitions from the crust to the filaments, suggesting filament growth out of the crust. This pattern has been observed for other endolithic communities in subseafloor basalts, specifically fungi, where the walls of the open pore space have been colonized by a biofilm from which filamentous hyphae or spherical yeast-like structures protrude and form mycelial-like networks [13–15]. The Vesteris Seamount basanites were colonized in a similar fashion based on the occurrence of crusts of secondary minerals and the morphological similarity with fossilized fungal biofilms [13–15]. The microbial colonization initiated by the biofilm formation occurred at an early stage and predates the ingress of marine sediments. Further, the occurrence of filaments in the marine sediments and their tight association with sedimentary particles indicates that microbial activity was contemporaneous with early ingress of seawater and subsequent sedimentation in the vesicles. The putative filamentous microorganisms are, thus, indigenous to the vesicles and syngenetic with the early infill of vesicles rather than being a modern phenomenon, postdating the formation of secondary minerals in the vesicles. The introduction of epoxy in the vesicles during the thin section preparation did barely disturb the fossilized communities. The indigenousness of the fossils is still apparent, since they are rooted on the crust at the vesicle walls or interwoven into the marine sediments.

3) *Morphology*. The occurrence of filaments organized in mycelial-like networks protruding from a crust lining the interior of the vesicle walls is identical to previous reports of fossilized fungi in subseafloor basalts [13–15] and is in accord with an earlier identification of the studied microtextures as fungi [20]. The overall morphology of the filaments and mycelial-like networks correspond to known fungal morphology [31]. Anastomosis between branches is a characteristic fungal morphology that excludes a prokaryotic interpretation. An inner strand is also seen among fungal hyphae and probably reflects shrinkage of the cell cytoplasm during fossilization or the lumen of a hypha with thick cell walls [12,31]. Size is not a reliable criterion to distinguish between prokaryotes and eukaryotes [32], but the overall diameter of the filaments ranging from ~5 to ~15 μm correspond very well to known fungal hyphae [31] and especially fungal hyphae from subseafloor basalts [12,13].

The oval terminal swellings of the first type of filaments correspond to hyphae with sporophores. In combination with the directed growth of the bush-like mycelia, members of the Eurotiales, an order of the Ascomycetes, are likely candidates [31]. Asexual reproduction among these fungi is by the formation of conidia on conidiophores, which match the shape and size of the observed swellings. Ivarsson et al. [12] interpreted similar fungal hyphae with chitinous cell walls as Ascomycetes or stem-group Dikarya from subseafloor basalts of an age of 48 Ma.

The second type of filaments observed in the basanites is much unlike the filaments of the first type. The occurrence of dark swellings along the filaments, swollen hyphal tips with mirrored morphologies and the presence of loops associated with these structures are characteristics that typify the Zygomycetes and its various sexual reproductive stages of diploid zygospore formation [31]. Here we use the class Zygomycetes in a traditional sense including the taxa Mucoromycotina, Kickxellomycotina, Zoopagomycotina and Entomophthoromycotina. Each of these taxa are significantly supported as monophyletic by molecular data but the relationship among them remains unresolved [33,34].

The Zygomycetes reproduction cycle is initiated by hyphae of different mating types approaching each other to form swollen hyphal tips, referred to as zygophores, which at contact develop into individual progametangia. Each progametangium is cut off from the original zygophores by a septum and separated into an individual gametangium. The two gametangia form a fusion cell, a zygote, which swells and develops a dark warty outer layer to become the zygospore. At the initial stages of zygospore formation two swollen, hyphal tips meet (Fig 4A) and form progametangia at this contact (Fig 4B and 4C). In Fig 4D and 4E the tips of the progametangia are separated by septa to form gametangia and the early fusion cell begins to develop into the still immature zygospore. Finally, Fig 4D shows a swollen, dark structure with a warty appearance that represents the last stages of the zygospore formation, the zygote. Among some Zygomycetes the hyphal tips of the zygophores get tightly appressed parallel to each other while fusing to form the progametangia. In this process a loop is formed (see Fig 4B and 4C). Based on all these detailed morphological characteristics, a continuum of different stages during zygospore formation is apparently present in the investigated basanites. Non- or irregularly septated hyphae (adventitious septa) further support the Zygomycetes interpretation rather than Ascomycetes or Basidiomycetes. Anastomosis among Zygomycetes is sparse but has been observed in several species [35,36].

4) *Composition*. Even though the hyphae were embedded in epoxy resin, distinct carbonaceous matter was detected by Raman spectroscopy in the zygospores. Comparisons with spectra of the epoxy exclude contamination and shows that the CM is indigenous to the zygospores. The localization of the CM to specific areas such as the zygospore core further suggest that only the central and protected parts still contain indigenous organic remains. Zygospores are produced to sustain harsh conditions, allowing Zygomycetes to flourish after long periods of environmental stress. The presence of CM in the core is, thus, in agreement with a sporophore interpretation.

The chemical difference between both the zygospores and the conidiophores of the tentatively identified Eurotiales-relatives on the one hand and the hyphae on the other hand probably reflects primary compositional variability between different organs rather than resulting from diagenetic processes. Reproduction organs like spores have a chemical composition distinct from hyphae. Both zygospores as well as conidiophores of Euriotales have high contents of melanin, and zygospores also contain significant amounts of β-carotene and sporopollenin [31,37]. The latter is extremely resistant to degradation and enables zygospores to remain dormant for long periods. A number of spore forming marine bacteria of *Bacillus* are known to enzymatically oxidize soluble Mn(II) to insoluble Mn(IV) oxides [38]. Enzymatic oxidation of Mn(II) to Mn(IV) has been observed in an *Acremonium*-like fungus belonging to the Ascomycetes, but was not found in association with its spores. Hansel et al. [39] have shown that Mn(II) is oxidized to Mn(IV) at the asexual reproductive structures of an Ascomycete species due to the production of extracellular superoxide during cell differentiation. The direct cause for the association of Mn-oxides with the Vesteris Seamount fossils is at this point difficult to deduce, but it seems obvious that the fungal spore formation was involved in Mn(II) oxidation and the formation of Mn(III,IV) oxide minerals.

The Vesteris Seamount fossils reveal that fungal diversity of subseafloor basaltic rocks is higher than previously assumed, introducing Zygomycetes as a member of the subseafloor biosphere. The abundant spore formation among the Vesteris Seamount fungi and the presence of an apparent reproduction cycle in the identified Zygomycetes indicate that the fungi were not randomly introduced by seawater but colonized this subseafloor habitat of interconnected vesicles in a sustainable fashion.

### Fungal metabolism in subseafloor basalts

Fungi are heterotrophs and the presence of fungal communities in depths of ~850 mbsf [13] in subseafloor basalts has raised questions regarding their metabolism. Possible sources of organic matter could be downward transport from overlying marine sediments, organic molecules produced from mineral-seawater reactions, or prokaryotic communities. Bengtson et al. [14] showed that fungi exist in close symbiotic-like relationships with prokaryotes at depths and suggested that the fungal metabolism is probably based on such interaction. The Vesteris Seamount samples represent a shallower environment of a few cms to dms below the seafloor, where marine sediments are abundant as vesicle fill. The fungal attack and degradation of the remains of marine organisms enclosed in the vesicles suggest that such organic matter is the major carbon source of fungi in these shallow basanites. The abundant small hyphae with diameters of ~1 μm are capable of penetrating the carcasses of diatoms and other marine organisms. They are interpreted to represent haustoria, specialized organs formed by hypha to penetrate inside carcasses to capture nutrients or used in parasitism. Haustoria are found among all major fungal divisions including Zygomycetes. The presence of haustoria indicates an adaptation among the fungi to scavenge on marine organisms. Even diatoms with their siliceous frustule were apparently degraded by the fungal haustoria, probably in combination with production of organic acids capable of dissolving the silica [40]. Obviously, fungi are early colonizers of fresh subseafloor basalts and ingress of marine sediments into shallow vesicles may provide the precondition for fungi to establish vital colonies in such an igneous rock environment.

## Conclusions

Highly vesicular basanites from the Vesteris Seamount in Greenland Basin contain fossilized cryptoendolithic fungal communities belonging mainly to Zygomycetes and subordinately to Ascomycetes. Zygomycetes has not been reported from subseafloor basaltic rocks previously. The taxonomic assignment is based on characteristic fungal morphologies including anastomoses, haustoria, conidiophores and zygospores. Among the Zygomycetes fossils a reproduction cycle has been identified representing different stages of zygospore formation. The spores have been mineralized as a Mn-oxide phase similar to romanechite. The hyphae, on the other hand, consist of limonite, a FeO(OH) * nH_2_O mineral. The spore structures have apparently been involved in Mn(II) oxidation and subsequent Mn(VI) formation, either enzymatically or by a matrix-solute interaction effect. The zygospores exhibit a central core of carbonaceous matter as revealed by Raman spectroscopy. Preservation of fungal organic substances was probably favored by the resistance of zygospores to environmental stress. The Vesteris Seamount fungi are closely associated with carcasses of marine organisms enclosed in marine sediments that ingressed into the vesicles. At the contact to carcasses, the fungi produced haustoria that penetrate into the carcasses and enabled the fungi to scavenge on the marine organisms. In volcanic substrates that are in close contact to sediments or exposed to seawater, like the studied basanites, marine organic debris may serve as the main carbon source, and facilitating fungal colonization and the establishment of vital colonies.
